# Vulnerable populations and the right to health: lessons from the Peruvian Amazon around tuberculosis control

**DOI:** 10.1186/s12939-019-0928-z

**Published:** 2019-06-03

**Authors:** Camila Gianella, M. Amalia Pesantes, Cesar Ugarte-Gil, David A.J. Moore, Claudia Lema

**Affiliations:** 1 Chr Michelsen Institute, Bergen, Norway; 2 Salud Sin Límites Perú, Lima, Peru; 3 Instituto de Medicina Tropical Alexander von Humboldt, Universidad Peruana Cayetano Heredia, Lima, Perú; 4 Tuberculosis (TB) Centre, London School of Hygiene and Tropical Medicine, London, UK

## Abstract

**Background:**

In 2014 the World Health Organization (WHO) launched the “End TB Strategy”, setting new ambitious goals for elimination of tuberculosis (TB). In contrast with previous efforts to control TB, the new strategy adopted the protection and promotion of human rights in TB prevention and care as a core pillar. This mandated the development of national programmes that are sensitive to the characteristics of populations and responsive to structural factors that put people at increased risk of exposure to TB, limit access to good quality health services and make people more vulnerable to TB infection. Indigenous people living in the Peruvian Amazon have been identified as a TB vulnerable group by Peruvian health authorities. This article examines the barriers faced by indigenous people and rural settlers from the Peruvian Amazon in obtaining a TB diagnosis and appropriate TB treatment, through the principles of the human rights based approach of accessibility, availability, affordability, adaptability and quality, and thus provides evidence of the utility of such approach in Peru.

**Methods:**

This is a qualitative study. We combined information from policy documents and legal regulations and in-depth interviews with health workers and health authorities. We used Atlas-ti to conduct a thematic analysis and identify interviewees responses to pre-defined topics.

**Results:**

Despite having a strong legal framework to protect the right to health of indigenous people and people affected by TB, there are underlying structural factors contributing to delays in detection, diagnosis and TB treatment, which are mostly related to having a health system poorly prepared to provide care for people living in dispersed rural communities. This article shows the limited level of integration of the “End TB Strategy” principles in the Peruvian National TB Programme and identifies the weakness of the health system to improve health care provision for indigenous people and rural settlers from the Peruvian Amazon.

**Conclusions:**

Our study shows the need to go beyond developing a strong legal framework to ensure vulnerable populations such as indigenous people are able to realize their right to health. Governments need to allocate funds, improve training and adapt healthcare provision to the cultural, geographical, and social context of indigenous people.

## Background

In 2014, the World Health Organization (WHO) launched the “End TB Strategy” setting new and ambitious goals for elimination of TB. In contrast with previous efforts to control TB, the new strategy adopted the promotion and protection of human rights as a key pillar for TB prevention and care. Adopting a human rights approach for tuberculosis implies the development of programs that are sensitive and responsive to structural factors that put people in increased exposure to TB, limit access to quality health services and make people more vulnerable to TB infection [1, 2].

However, despite formal engagement towards human rights on TB prevention and control, there are concerns regarding the limited commitment of countries to implement a rights-based approach to tuberculosis control. As a result, external evaluations have stressed that *current levels of progress fail to meet state obligations to progressively realize the right to health and are insufficient to meet global TB targets* [3]. This constitutes a call to generate evidence of the adoption of a human rights based approach by states, including high burden TB countries like Peru [4], as well as evidence on the steps taken to address the needs of vulnerable populations whose right to health is not being fully realized. Such is the case of indigenous people in Peru.

Peru is an upper-middle-income country (with a gross national income (GNI) per capita of US$11,295), yet 20% of the population lives in poverty [5, 6]. Disparities in the distribution of wealth – the inequality adjusted human development index is 0.585 – are closely related to ethnicity. Although it is difficult to establish a single marker of ethnicity in Peru, one common proxy is mother tongue, which enables identification of the inequalities faced by indigenous people. For instance, while 41% of people with an Amazonian language as their mother tongue have an annual income below the Peruvian poverty line of income, the national average is 8% [7]. The Amazon region, and rural areas also report higher level of illiteracy. Tuberculosis (TB) is a disease closely associated with poverty and Peru is no exception [8, 9]. For example, in the Peruvian Amazon, the regions with the highest percentage of indigenous people (Loreto, Ucayali and Madre de Dios) are regions with very high TB incidence rates (99, 145 and 129/100,000 population/year respectively). The Junin region, where we conducted this study has also been rated as a high TB risk region (incidence rate of 51/100,000 in 2017) [10].

Here we report the results of a study conducted among indigenous and rural communities in the Peruvian Central Amazon to understand the limitations in TB service provision through a human rights lens. TB services include the process of detecting potential TB patients, diagnosing those detected and treating those diagnosed (DDT). This paper aims to provide evidence of the significance and validity of using a human rights approach to assess the performance of National TB programmes. We do so by using the human rights framework to identify the challenges and implications of the provision of TB diagnosis and detection in a particularly vulnerable population in Peru [11]: the indigenous population living in the rural and geographically dispersed communities in the Peruvian Amazon (mostly of the Ashaninka ethnic group). We specifically focus on the human rights principles of acceptability, availability, affordability, adaptability, and quality (adapted from the AAAAQ- criteria), essential elements of the right to health [12] to analyse the detection and diagnosis processes. The focus on these two components (detection and diagnosis) is not arbitrary, but rather it responds to a national and international concern regarding underperformance in these domains [2, 13].

Secondly, the article aims to assess the degree to which Peruvian public health policies are sensitive to inequities in access to health care and health needs, and whether they are designed to contribute to reducing those inequities faced by vulnerable populations. Pursuing health equity involves a commitment to minimize the social determinants of health that increase the risks of getting sick, facing delays to access adequate and timely treatment; and reflects the respect of human rights principles [14, 15]. There is no agreement on how to assess the level of penetration of human rights principles in National TB Programmes. However, as a minimum it is important to have a more thorough discussion to determine if current TB indicators are suitable to assess and provide information on the respect of human rights principles by National TB Programmes.

### The right to health under Peruvian law

Peru has committed to protecting and guaranteeing the right to health for its indigenous populations, as well as for people affected by TB. As we will see, Peru has made efforts to develop a legal framework as well as policies to ensure comprehensive health care for indigenous people of the Amazon, and people affected with TB in general. However, there still is room for improvement.

Domestic legislation regarding the protection of the right to health dates to the 1993 Constitution. This Constitution is characterized by a limited development on fundamental rights. For instance, it does not include the Economic, Social and Cultural Rights as fundamental ones. Therefore, while it recognizes the right to ethnic and cultural identity as a fundamental right (Article 2), the right to health is under the non-fundamental group of rights (Article 7). The 1993 Constitution also establishes the State’s obligation to respect the cultural identity of indigenous communities (Article 89), which provides the platform to ensure health services are culturally appropriate.

On the other hand, the 1993 Constitution states that international treaties are part of national law (Article 55) since international treaties have constitutional status [16]. In addition, the Fourth Final and Transitory Provision of the Constitution (*Cuarta Disposición Final y Transitoria*) states that “the norms relating to rights and liberties recognized in the Constitution are to be interpreted in accordance with the Universal Declaration of Human Rights and the human rights treaties and agreements that have been ratified by Peru” [17].

In the field of regulations, the Peruvian General Health Law recognizes health protection as an element of public interest and public responsibility of the State [18] (Table 1). The same law states that the management and regulation of actions to prevent, control, and eradicate communicable diseases throughout the country fall within the responsibilities of the national health authority. In 2014, the Law for TB Control and Prevention (Law 30287) was passed, and it includes, among the rights of people affected by TB, the right to receive complete treatment (prevention, diagnosis, treatment, rehabilitation, and where required, specialized care according to the national TB Guidelines). This law’s regulation (issued on 2016), does not mention indigenous peoples or the need to provide culturally adequate health care to people affected by TB [19], highlighting the limited effort invested to ensure alignment and coherence between general laws and sectorial regulations.

**Table 1:** Peruvian Law and the Right to Health

Law or Regulation	Recognizes Health as a Human Right	Recognizes the need to provide special care for indigenous people
Constitution 1993 (with Jurisprudence of Peruvian Constitutional Court and Fourth Final and Transitory Provision of the Constitution)	X	x
Peruvian General Health Law	(health protection)	
Law for TB Control and Prevention	X	
Peruvian National TB Guidelines		x

The recognition of indigenous Amazonian peoples as a TB-vulnerable group is clear in the 2013 Peruvian National TB Guidelines. This document, issued by the Ministry of Health, recognizes the need to create synergies between the health systems of indigenous people and the public health system which is based on a biomedical approach, and the need to provide TB care using an intercultural approach [11]. Unlike other guidelines issued by the Peruvian Ministry of Health, such as that for HIV, Peruvian TB guidelines do not include any recommendation about special measures to be taken in rural contexts or in dealing with isolated communities, which is where most Amazonian indigenous people live. This is not a minor observation, because one core characteristic of health services in isolated and rural communities is the lack of access to complex health services offering for example TB culture tests, chest radiography, clinical assessment by a medical doctor or evaluation by a psychologist or a social worker. All these procedures are included in the Peruvian National TB Guidelines for TB diagnosis and prior to initiation of TB treatment. In rural isolated communities only basic primary health services are offered; such settings are not equipped for the analysis of sputum samples or for blood tests. Healthcare is usually provided by health workers with technical degrees, such as nurse technicians or midwifes.

In Peru, all low-income people are entitled to free enrolment to the public health insurance plan (*Seguro Integral de Salud - SIS*). Yet, until 2014, due to multiple administrative barriers, indigenous people from the Amazon were unable to access SIS and other governmental social programs. To address this problem the Peruvian government issued, in October 2014, a package of executive decrees, which required, inter alia, all inhabitants of indigenous Amazonian communities to be classified as a population living in extreme poverty, and consequently to automatically qualify as beneficiaries of all social programs and SIS. This was an important step to secure the right to health for indigenous people. However, as our research shows, this is far from being enough. This paper explores to what extent the legal framework to ensure access to TB care for indigenous people is being implemented through the Peruvian public health system.

### Peruvian national TB program

Despite being a pioneer in the implementation of directly observed treatment, short-course (DOTS) for TB control, the national TB incidence and prevalence in Peru largely exceed regional levels. Peru has the second highest incidence of TB in the Americas [20], with more than 40% of the multidrug-resistant TB (MDR-TB) of the Americas (but only 3% of the population). The World Health Organization (WHO) has listed Peru among the countries with the highest burden of MDR-TB worldwide.

As in other high-burden countries [21], one of the weakest areas of the Peruvian National TB Program (PNTBP) is the timely detection and diagnosis of people with TB. According to the WHO approximately 16% of cases are missed, meaning they are either not detected, not diagnosed or not notified [4], allowing the continuing spread of the disease within communities.

There are several health system factors that contribute to delays in detection and diagnosis. One factor is the lack of knowledge about TB among the population (only 3.4% of the population over 15 years old have heard about TB and recognize how the disease is transmitted) [22]. There is also an important gap in contact tracing and implementation of preventive measures for contacts of people affected by TB [23, 24]. Only 66% of under 5 year old child contacts of bacteriologically confirmed index TB cases receive preventive TB therapy [4]. This underperformance has been linked to the lack of resources for health workers to do home visits [25].

The microscopic examination of sputum samples has been and continues to be the main diagnostic tool for TB among people over 15 years in Peru. The national guidelines require that persons to be evaluated for TB (over 15 years old) provide at least two sputum samples, collected at least 30 min apart. There is guidance regarding the quality control of the samples, including the quantity (at least 5 ml), conservation and transportation. According to the guidelines, the samples must be evaluated at the local laboratory, and this must deliver the result within the 24 h of reception of the sample. All people with a positive TB result must undergo a drug susceptibility test to detect MDR-TB and a culture. Cultures are used as a diagnostic tool, but also as a means of quality control for peripheral laboratories.

TB services, including diagnosis and treatment, are provided for free at public health facilities. People affected by TB that do not have health insurance are entitled to a free health insurance plan (SIS). The network of public health facilities administered by the Ministry of Health (MoH), has national coverage. The MoH hires around 70% of the health workforce, and has the strongest presence in rural and dispersed communities.

Health workers from public health facilities must perform both passive and active-case finding of TB. This means they should detect person with presumptive TB (i.e. people with 15 days or more of cough) among the population visiting health facilities (passive case-finding). Furthermore, health workers are charged with the organization of outreach activities with groups that have been identified as high risk for TB transmission and outbreaks, such as prisons, the homes of TB patients and rural communities (active case-finding). The national TB guideline states that TB detection and diagnosis cannot be performed as part of massive health campaigns among asymptomatic populations.

The MoH has quantitative targets for case detection and diagnosis per health facility. These targets are defined by the central government and reported by regional authorities in order to determine funding allocation for that region (*Presupuesto por Resultados*, “Budget for Results”). Under this scheme, it is expected that health facilities identify and screen 5 individuals with presumptive TB for every 100 consultations with people over 15 years old [26].

## Methods

### Setting

**Fig. 1:**
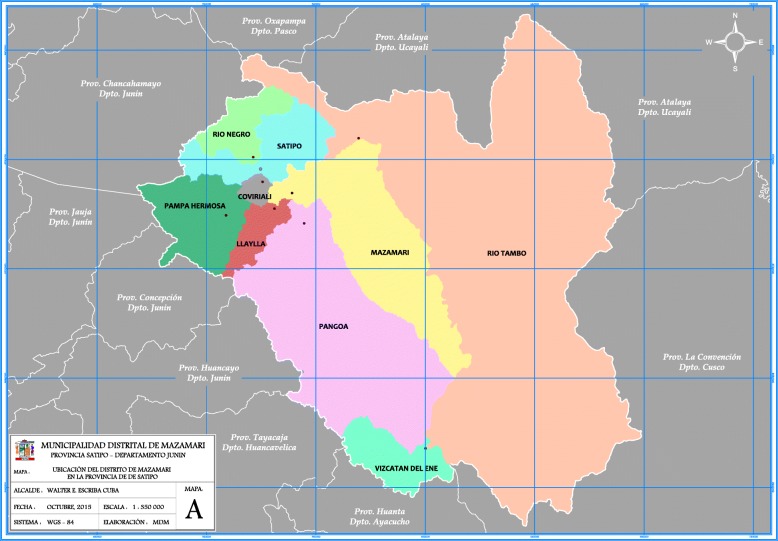
Provincial Map of SatipoAuthor: Municipality of MazamariDate: October 2015

Data was collected in the districts of Pangoa, Rio Negro and Satipo, in the Junín region (see Fig. 1 below, Map of Satipo. Source: Municipality of Mazamari). Junín region is multicultural and multilingual. Peru has 55 indigenous groups, 51 from the Amazon region. Amazonian indigenous communities occupy around 44% of the region’s territory. Nine of the 13 districts of Junín (total number of districts = 123) for which the majority population are dispersed Amazonian indigenous people, are in Satipo province. Around one third of the population in Satipo lives in poverty [27]. The Ashaninka are the most numerous Amazonian Indigenous group in Peru, and the majority of them are settled across Junín Amazonian districts.

Originally, we planned to focus the data collection only on Ashaninka indigenous populations and communities, in the district of Pangoa, mainly along the Ene River. However, during interview with the manager of the TB programme in Satipo in September 2016 it became clear that the challenges faced by the TB programme in indigenous rural and dispersed communities were similar to those in non-indigenous small towns and non-indigenous rural communities, regardless of the ethnicity of the dwellers. Besides, the itinerary that indigenous people must follow after being diagnosed with TB implies travelling to health facilities outside their communities. As a result, we decided to include non-indigenous rural communities in the study.

Communities along the Ene River are located in a coca production area, with consequent presence of military forces and drug-traffickers. People living in several of the non-indigenous communities are seasonal workers involved in illegal logging or harvest and transport of coca. Satipo and San Martín de Pangoa’s health directorates have mainly health facilities from level I-1 (staffed only by a nurse technician and maybe another health worker) and level I-2 (health post or health centre with a medical doctor or midwife and a nurse technician). There are two referral hospitals, one in Pangoa and one in Satipo.

### Study design and data collection

This study used a qualitative approach. This manuscript presents the results of the analysis of health workers, and TB program managers´ interviews. These actors were selected since they were the ones who provided insights that allow us to understand the characteristics of the health system and the challenges of implementing TB health policies and regulations in a rural context. All interviews were conducted in Spanish and at the workplace of the informants.

Interviews were undertaken between September 2016 and January 2017. The selection of health workers was based on accessibility; this included security considerations for the research team. The following exclusion criteria were applied in determining site selection. We did not collect data in communities that: (1) required more than 4 h walk to be reached, (2) had experienced recent violent events (clashes between the community and the drugs traffickers), (3) were known to harbour a prominent presence of drug traffickers and other illegal activities (like illegal logging). Sampling was performed in identified sites until saturation. In the case of TB program managers, we included all TB program managers in the study area. We completed 19 in-depth interviews with health workers from 12 health facilities including lab technicians from the reference laboratories at Rio Negro, San Martín de Pangoa and Satipo. We also interviewed the TB programme managers from San Martín de Pangoa, Puerto Ocopa, Rio Negro and Satipo and the regional health director from Junín region (five in total).

Interviews were performed at health facilities on the day and time interviewees indicated it to the research team. Interviews were performed by trained members of the research team (psychologist and anthropologist) with experience in qualitative data collection methods with the same type of population.

### Data analysis

Recorded audio data from individual in-depth interviews of the four aforementioned actors were subjected to careful transcription. Transcripts were read and re-read, following thematic analysis, searching for emergent themes and recurrent ideas. Thematic analysis is a suitable method for examining the perspectives of different research participants, highlighting similarities and differences, and generating unanticipated insights [28]. All transcripts were then coded using AtlasTi-8 software program (Scientific Software Development GmbH, Germany) using a codebook created according to the emerging themes. For this paper, we used those codes that allowed us to extract and organize the data according to the two key elements of the provision of TB care explored in this paper: detection and diagnoses.

### Ethical consideration

Ethical approval was secured from the Ethics Committee from Universidad Peruana Cayetano Heredia. Approvals from National TB Programme, Junin regional health authorities and Satipo health directorate were obtained before the study started. Written informed consent was taken from participants including for audio recording. Alphanumeric code numbers were assigned to the interviews and transcriptions to ensure participant confidentiality.

The research project was also presented to indigenous organizations. As part of the project were organized workshops to return the main results to health authorities and indigenous organizations.

## Results

### TB detection

When we asked health workers to describe their efforts for active case finding of TB suspects, they immediately began talking about the targets they had to reach. Reaching such targets appeared to be the main driver of their efforts. We explored the problems faced by health workers to reach the National TB Program goals, specifically the target related to the testing of TB suspects, and realized that the numeric goal (i.e. the number of TB tests performed) has wrongly become the main objective: health workers directed all their TB-related efforts to accomplish such numeric goals, including applying the test to individuals who would not be considered TB suspects, sacrificing the rationale of the procedure.

*On a monthly basis we have to show (provide information) on the targets annually established by the Health Directorate. For example, they tell us that this year we have to get a certain amount of samples and they divide them to give us a monthly target. For example, “this month you have to get 36 samples.” That is a target, but sometimes we cannot meet it so we have to go and search for those patients who are respiratory asymptomatic, but still we do not reach the target (TB nurse, Interview 16).*

During our fieldwork, we saw that health workers tested people not suspected of having TB. For example, they performed TB tests on people seeking a health certificate for work-related purposes. These tests were then reported as testing of a TB suspect. Health facility managers accepted this practice: they even supported advertisements for free TB testing for those in need of health certificates.

One of the main barriers identified by healthcare workers and programme managers to achieve the targets is the lack of human resources. This is connected with financing. Healthcare workers, especially those working in rural health facilities, have to spend many hours travelling to visit the communities within their jurisdiction. Furthermore, they are overburdened, since each worker has to oversee several health programs, (i.e. vaccination, sexual and reproductive health, malaria) which means preparing one monthly report per program. The lack of human resources and health facilities translates into reduced accessibility and availability of opportunities for case detection for people living in rural communities like the ones visited in this study in the Peruvian Amazon. In order to receive TB detection procedures, this population needed to wait until a health worker visits their community, since most do not have the resources to travel to health facilities.

*The community of Shempimtiari is an hour and a half away walking - that is what it takes the inhabitants. Now if you want to go to Puerto by car you have to wait 1 h, from there you have to go to Sanitie half an hour or 45 min, from there to Pampa Alegre you have to go 1 h walking, from there to Valle Kempiri, it is connected but far. Imagine a health worker that goes there, it takes 3 or 4 days, and there you have to do everything. I do not know what malabares [juggling moves] he will have to do to provide care in all the areas; I do not know how he will be doing the patient recruitment. Then when he comes after 3 or 4 days, he arrives at his health post, sometimes he has to fix [the sputum sample] there or sometimes in Pampa Alegre but if they bring a sample, sometimes they do not always go to the communities, because they are only required to go once a month (Nurse technician, TB manager of one health network Interview 5).*

**Accessibility** to TB case detection was also negatively affected by the lack of commitment and awareness of health workers who are not in charge of TB services. This lack of commitment translated into overlooking TB suspects that reach their service, which in turn, affected the timely detection of TB, suspect cases among people that reach health facilities looking for help.

*The doctor does not detect [potential TB patients], he is the one who should detect the most, yet only Mr. Pedro and I detect patients. Sometimes we go out to look, we go to the home visit to get the patient, the patient is ashamed [to be identified as potential TB patient] to produce a sample. (Nurse technician, TB manager of one health network Interview 5).*

*I was passing by [the doctor’s office] one day and she was prescribing fever [medications] and I asked the patient, just in case: “Young man, for how many days you have been coughing?” and we go [to make a test] and it was positive. You can imagine [what would have happened] if she gave her treatment [for the fever], he would have gone away and returned three months later with a higher bacilli load. There are few who support us, not all of them… (TB nurse technician Interview 1).*

**Fig. 2:**
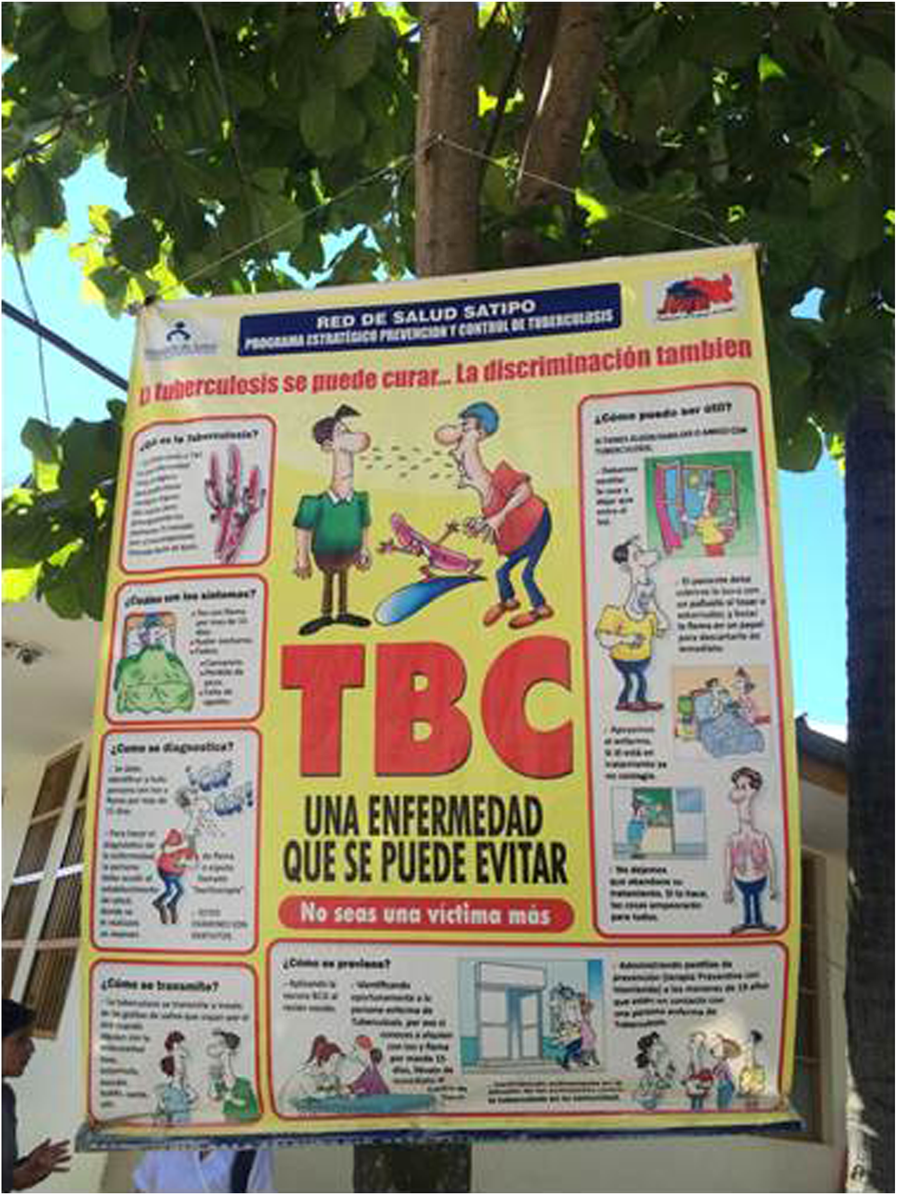
Example of Educational material produced by Satipo’s Health Directorate. The material targets inhabitants of the Amazon region, however shows information and drawings referring to an urban setting in the coastal region Author of the photograph: Camila Gianella.

**Fig. 3:**
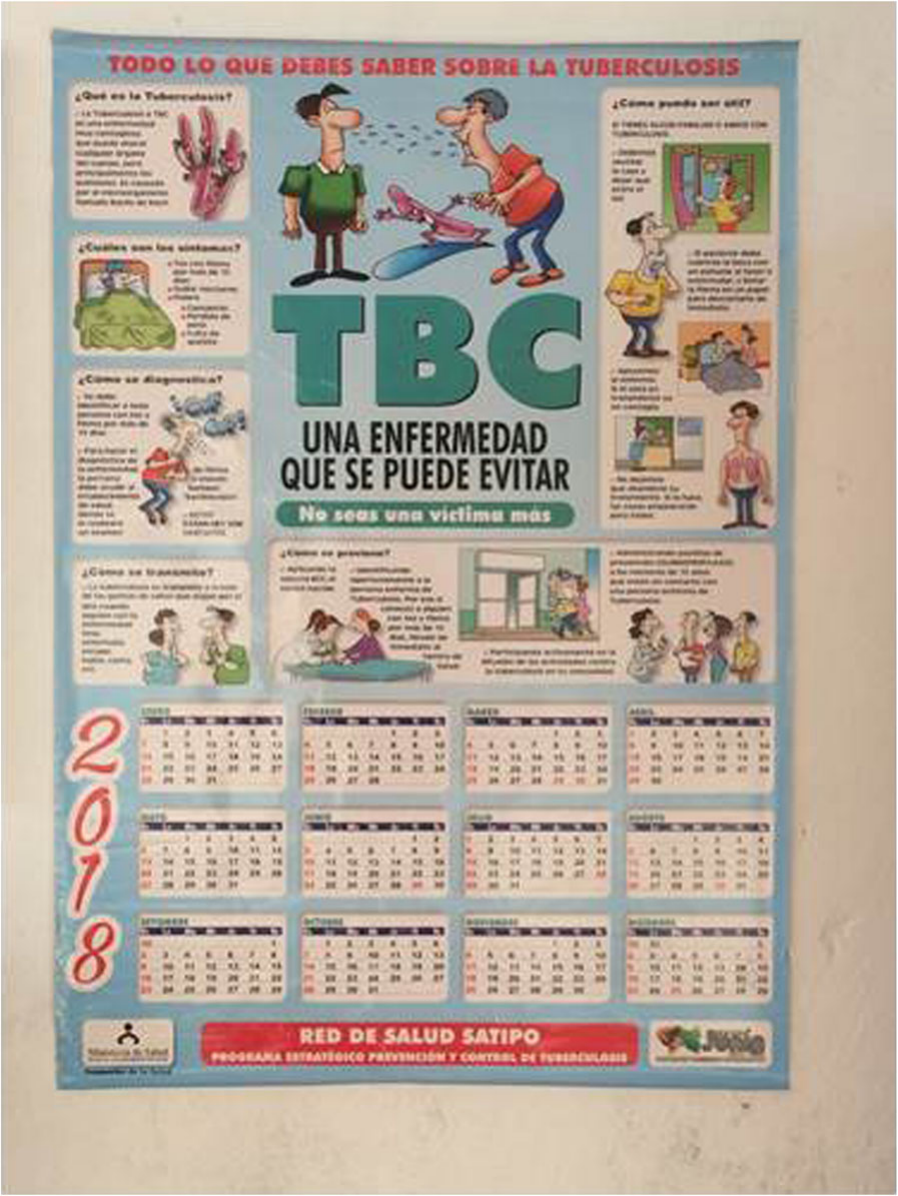
Example of Educational material produced by Satipo’s Health Directorate. The material targets inhabitants of the Amazon region, however shows information and drawings referring to an urban setting in the coastal region Author of the photograph: Camila Gianella.

When exploring the cultural adaptation of the services and of the information provided, we learned that most of the information at health facilities is in Spanish and designed for urban settings (Figs. 2 and 3 show information material exhibited at Satipo and Pangoa hospitals). This happens despite the fact there are national guidelines that were developed in 2015 to train health workers on how to work with Ashaninka indigenous communities with an intercultural approach [29]. Healthcare workers that could benefit from these guidelines are not aware of them and have not received special training. This not only happens with TB; the MoH does little to prepare their own healthcare workforce before sending them to indigenous communities. The adaptation to the new setting with a new culture and language was described as a challenging experience for health workers who often come from other parts of the country, usually from urban settings.

*At first, it was shocking, they speak an [indigenous] language and I speak Spanish and also Quechua and we did not understand each other. The one who helped me the first few years was my health promoter - I thank him for that. Thanks to him I learned many things from the community. He taught me the language, when he took care of patient he sat down and he translated, then in the hours that there was no patient in a sheet of paper he taught me some words and so now I understand all that, and I speak a little. (TB nurse technician Interview 17).*

Another issue was that most of the health workers in charge of delivering TB care to patients affected by TB are nurse technicians (*técnicos de enfermería*) and nurses. They stated a need for more training to those in charge of the daily deliver of TB care. Although we were told that trainings are regularly organized, the problem is that usually physicians or people with administrative roles are the ones invited, neglecting those in charge of direct attention and patient care.

The focus on numeric goals, lack of financing for proper information campaigns, lack of culturally adequate material, limited training on intercultural skills and on TB in general affected the quality and impact of the TB detection effort. Detection of the truly suspected cases in the communities has become a passive activity, and health workers only react to perform an adequate TB active case finding when there are outbreaks, missing the opportunity for early case detection and to cut the transmission of the disease at community level.

*The work, in fact all the health personnel of the 25 establishments, including the health centre, we make a search, I would lie if I say active - it is almost passive. One in the periphery and here it becomes passive, because when it comes to the symptomatic when it comes to the establishment, let’s just say that we put the energy and do an active search when there is a case, or many times when they refer to us from another establishment. (TB nurse, Interview 7).*

### TB diagnosis

TB diagnosis involved two procedures, 1) TB detection, mainly performed through the microscopic examination of smear sputum samples, and 2) sputum TB culture, applied to all people with a positive TB smear microscopy test; this must be performed before starting treatment (because it is necessary to identify a potential patient with MDR-TB).

Key elements of the access to TB diagnosis is the availability of supplies to allow the adequate collection and transportation of the samples, as well as the availability of a laboratory network that allow samples to reach the reference laboratory in adequate condition for proper, and timely examination.

Long distances to the laboratory facilities has forced the implementation of an informal delivery system: health facilities closer to the reference laboratories sent fresh samples to the laboratory, and health facilities from remote areas, sent smears on microscope slides that have been previously heat-fixed at the laboratory.

*Yes, the health posts send the samples [to the reference laboratory] downriver that have already been heat fixed. Some posts that are close to us send the fresh samples. The health posts that are further away send us the samples that we have already taught how to heat fix. We train them and they bring us the sample so that we can stain them and we ‘read’ them. (Lab Technician Interview 12).*

The first major barrier faced by health workers from remote health facilities is the lack of supplies. Health workers working at basic primary health facilities (level I-1 health facilities in the Peruvian health system) in rural areas are required to fix the smear sample on a microscope slide with heat, but they did not have access to Bunsen burners. Thus,they used candle flames to fix the smear material; an inappropriate way to prepare the smear. This procedure not only goes against the National TB Guidelines but constitutes a hazard for health workers, and does not guarantee the proper management of the sputum samples.

Distance also constitutes a barrier. Long distances and thus the time to take the sample from where it was taken to where it can be analysed, affects the quality of the sample as well as diagnosis turnaround time. Health workers from isolated areas only sent the smear samples once a month, meaning that, in these communities, it can take up more than a month between the moment the sample was collected and the results arriving back at the health facility. The reasons for these delays are mainly financial. There is not a regular system for transporting samples in a safe and timely fashion. Samples are transported and delivered by health workers during their monthly visits to the head of the program. In other cases, samples are sent by public boat.

*The diagnosis, if we talk about the hospital, takes 24 h. In extreme cases, 12 h. In San Ramón and Sonomoro where they have their laboratory, if the sample reaches the health center it will also be 12 to 24 h [until the results are out], but to get there, the health post, for example Santa Elena will take at least one day, diagnostic tests one more day and then, go back and forth, one more day. Patients who are not positive could be infected. Cubantia… we would be talking about 4 days, 5 days. Every 20 days they come downriver [to the reference laboratory] and sometimes they send out the slides with the samples alone, and sometimes they can get lost. (TB nurse technician Interview 1).*

***When you pick up the sputum test, you tell me you usually heat fix it here?***

*Yes I fix it here.*

***More or less how often do you send samples to Puerto Ocopa? (local reference laboratory).***

*Every fifteen days or nine days I have to be sending [some samples], depending on the amount.*

***Do you prepare malaria slides here, too?***

*Yes, also malaria…*

***And you send it every fifteen days, and how do you send them, do you take them or do you send them with the boat?***

*I send them with the boat.*

***And do they arrive normal [on time] or is there a problem with that?***

*Sometimes they get lost, and we have to pay five soles to take them.*

***Is it sent using resources from the health centre?***

*No, we pay it with our own money; out of pocket of our work.*

*(TB nurse technician Interview 3)*

Another major problem is the quality of the samples. Not all health workers collect good quality samples, and some samples get damaged during their transportation.

*Well, there are posts that bring you good quality samples, there are posts that try to bring the samples that they consider are pathological but with the movement the samples arrive already liquefied. Other health posts bring us saliva samples, there are many saliva samples, their incidence has increased in recent months, they are more salivary samples than mucous. (Lab technician Interview 12).*

The poor quality of the samples has been linked to the high level of rotation of the health workers, including laboratory technicians from rural health facilities.

*Each time as I say, sometimes the problem we have is that we train the hired staff, then something happens and the next month we have a new staff and does not know [how to take samples], suddenly and have no interest in requesting training or in any case to come.*

*We tell you, if you do not know, come over here, I will not tell you, we will not teach you, we will indicate what the measures are two by one, how much you should use as a sample, how you should process it. That is for us the quality control, because in reality [if one makes a mistake], we are all dragged with the mistake, if they don’t fix it well. We also do poor quality control, of what use is it that here we do all the right measures, as it should be done, if they do not practice that? (Lab technician Interview 11).*

Lack of human resources also affects the time of processing samples, and therefore the diagnosis, delaying the start of treatment.

***And that [the sputum sample] is read here?***

*Yes, [name of the Laboratory technician] is in charge of doing the reading.*

***But now [name of the Laboratory technician] is not here?***

*Yes, if he [referring to the laboratory technician] is not here, the samples arrive at my house, they look for me and gave me the TB and malaria samples.*

***How many days will he be away [name of the Laboratory technician]?***

*Four days, because it’s this months’ time-off. He gets 10 days rest, because they are 21 days of rest, and they distribute them like that.*

***And when he leaves there is no one who reads the samples?***

*No, when he leaves, we will not take samples in several days. I think these samples have been here one day, how many days they might have been in their posts, then sent here, until [name of the Laboratory technician] arrives and then the result are returned, imagine how much time has passed. (Nurse technician, TB manager of one health network Interview 5).*

For the diagnosis of MDR TB and for sputum cultures, patients must travel to Satipo or Pangoa. The cost of the transport was covered by patients, and treatment did not start until they had gone through baseline tests and gotten an order issued by a medical doctor to start the treatment. Many (infectious) smear-positive patients have no choice but to travel in public transportation for several hours to reach a health facility where they can be examined by a medical doctor and start their treatment, coughing during the trip and increasing the transmission to the community. This creates delays to the start of their treatment, affecting both TB patients and the community. In many cases health workers get involved in coordinating with community authorities and local authorities searching for the resources to send the TB patients to Satipo and Pangoa. This is something that TB program managers are aware of; however they did not have a solution.

*[First] you have to go to Satipo and then the treatment starts here. We have to take the cultures. And just in the case of [name of TB patient], it was one of those cases that came out positive and had to go down [Satipo] to have their cultures taken, we had to wait, it is not fast, we all thought it was simple TB, but in the end it was MDR. (Nurse technician, TB manager of one health network Interview 5).*

In addition, there were also delays in the delivery of results. In the case of MDR-TB the lack of access to computers and internet at the rural health care facilities, prevents health workers from having access to the results of the TB patients under their care.

All these findings highlight serious weakness in several areas of the Peruvian health system. This situation is complicated to resolve; most of these health centres are remote, rural or semi-rural and underfunded, most of them cannot afford the appropriate information technology (IT) to evaluate their data in order be more efficient providing TB care. One situation the research team observed and discussed during the data analysis process an issue that deserves further research: what are the implications of testing asymptomatic people who request a sputum smear test for reasons unrelated to TB diagnosis, for example persons seeking health exams for work, or couples who are getting married because is requested by the mayor’s office. Such cases amounted to more than 25% of the samples tested during the year, both artificially inflating the diagnostic testing metrics and potentially distracting from diagnostic efforts by clogging the system with unwarranted testing. A simple but efficient IT program could help to identify these issues and can help in the improvement of TB care. There are some first steps from the National TB Program such as the launch of the SIGTB platform [30] as an operational database that includes all TB patients in the country, though it is likely that more tailored solutions are needed for areas like Satipo.

## Discussion

Our results show that although Peru has created a series of policies and regulations that aim to guarantee the right to health i.e. to ensure the accessibility, affordability, availability, adaptability and quality of health care for all Peruvians, we are falling short of implementing them. Translation of policy into practice is often challenging, and those challenges are further amplified in socioculturally and geographically marginalized populations. Despite health not being listed among the fundamental rights by the Constitution, Peru has shown a formal commitment towards the protection of vulnerable groups, including indigenous people. Indigenous people from the Amazon have been officially recognized as a TB vulnerable group, and there are public policies in place that ensure their free access to health care through the SIS, but the health system is affected by several structural factors that jeopardize the realization of the right to health for indigenous people. The weakness of health systems has been recognized to affect the implementation of human rights; an ineffective health system does not guarantee the fulfilment of the right to health for patients. [31]

Our study shows that using the human rights principles of acceptability, availability, affordability, adaptability, and quality (the AAAAQ criteria) to analyse both the performance of the health system and its current assessment indicators affords useful key insights for further improvements. For instance, the dispersed distribution of health facilities, the poor organization of the public laboratory network, deficiencies with human resources provision and retention, and management for rural and dispersed areas, and general financing of the health system, create barriers to delivery of equal access to health services. As other authors have argued, an effective and integrated health system is crucial for securing the right to health of vulnerable populations [32]. Such a health system should encompass medical care and the underlying determinants of health, to ensure it is responsive to people’s priorities and accessible to all [32]. The structural factors of the health system we have documented are negatively affecting the impact on TB control, contributing to the dissemination of the disease amongst both indigenous and non-indigenous communities living in isolated and rural communities of the Peruvian Amazon, violating their right to health [33].

Our findings also provide evidence on the negative effects of segmenting health services by programs. Health workers are evaluated independently according to the performance of the programs for which they are responsible. We observed that in health services where programmatic responsibility is distributed among healthcare workers, such as health units of higher complexity (health centres and hospitals), healthcare workers did not feel a sense of ownership or belonging for the programs that were not under their direct responsibility. Therefore, only health workers directly involved in TB are taking responsibility for the detection of TB cases, and TB cases are missed, even when people reach the health facility searching for health care. This is contrary to the National TB Guidelines which states that the identification of persons with TB symptoms is a responsibility for all health workers (including those with administrative duties). Certainly, in Peru, TB is still a highly stigmatised disease, perceived as a disease with high risk of infection and this contributes to the lack of engagement with TB related activities. Our research found that apart from workers directly involved in the daily care of TB patients, most health workers are not sensitized to the importance of detecting TB cases. This situation also creates overload of work on TB staff because TB detection goals are designed within a conceptual framework in which there is a shared responsibility among the whole health facility.

In the case of TB indicators our analysis shows how the emphasis on quantitative goals, without a proper quality control mechanism to guarantee that the tests are applied to TB suspects, is resulting in an inefficient use of resources (human and supplies), in a setting where resources are scarce. A side effect of this practice is that it is impossible to know the real epidemiology of TB, and to improve strategies for active case finding. Putting pressure on performance for health workers has shown mixed results for improving the quality of care [34]. In our study, this pressure affects the possibility of really finding active cases of TB among indigenous people. Our results show how the focus on quantitative and measurable goals, and the lack of resources across the health system, have incentivised paradoxically harmful practices such as the testing of non-TB suspects. The results also show how, in a system focussed on some performance indicators, simple red flags of laboratory quality control, such as a low proportion of positive cases amongst those tested, are ignored or missing in quality control evaluations. Thus, current indicators to assess the performance of the TB program are not sensitive to these structural factors, and do not detect underperformance of Peruvian NTP. Despite the evidence regarding the failures fulfilling the goals of TB suspect detection, the reports are broadly accepted by health authorities and goals are, formally at least, fulfilled.

Our findings suggest that there are some key indicators that could work better to show inequities in the performance of the NTBP; for example comparisons between urban versus rural settings or concentrated versus dispersed populations. Metrics of interest could include the time taken for the analysis of smear samples and the time taken for the delivery of TB results, which are currently not part of the regular performance indicators of NTBP. Other key indicators should be the time between the delivery of the positive test results and treatment initiation. Most of the current TB indicators are not strong enough to assess if NTBP are sensitive and respond to structural factors that put people in increased exposure to TB, limits access to quality health services and make people more vulnerable to TB infections. Highlighting inequities in the health system performance is a useful first step to secure non-discriminatory access to services [35–37].

Another key finding is related to the adaptability of the National TB Guidelines to rural settings. The National TB Guidelines provides a framework for the information flows, procedures and responsibilities. In a country like Peru, characterised by topographic and cultural diversity, there are major differences regarding the capacity of the health system, the availability and accessibility to complex health units, and the characteristics of the tasks performed by health workers with the same level of responsibility. National TB Guidelines should reflect this diversity and include strategies to guarantee an adequate and timely TB case detection and diagnosis. Health workers interviewed in this project described different work settings, with different challenges (like distance to the communities, cost of transport or access to supplies) and local resources (such as the engagement of traditional authorities, support from indigenous leaders, presence and support from community health workers). However, this diversity is not reflected in the national TB guidelines, because the guidelines were planned for areas with high TB incidence like big cities such as Lima. It should be a key concern that national health programs, as the TB program, must better recognize the country diversity, the different context in which health workers work, which is central for taking decisions like who must be invited to the trainings. This research showed that in a rural setting as the one studied by this project, technical staff, as technical nurses, are the ones taking most of the responsibility of TB programs, but nevertheless they are neglected from trainings.

Another central point showed by this research is the risk of adopting a vertical segmented intercultural approach linked to specific programs, like maternal health, and not a comprehensive intercultural approach involving the whole health unit. This is an issue that needs further study and reflection, however we found that in a Level 1 health unit (*posta de salud*), where the same technical nurses oversee all programs and services provided at the health unit (maternal health, children development, malaria, dengue), they have the tools and indications to provide culturally adaptable maternal health services, but no indications on how to provide an intercultural approach in the case of TB. Intercultural approach is the right of indigenous people to receive health care adaptable to their visions and knowledge, in that sense, the health service as a whole must adopt an intercultural approach, and national TB guidelines must be adaptable to these realities.

Our study also calls for a reflection on how policies, both at national and international level, are addressing TB. An excessive focus on the national indicators (like national TB prevalence and incidence), without an analysis of vulnerability, or avoiding an equity approach, could leave behind the vulnerable and historically marginalized populations, such as the indigenous from the Peruvian Amazon, excluded from national statistics and policy efforts. The most recent emergency plan for tackling TB in Peru only included the city of Lima and four regions (Callao, Ica, La Libertad and Loreto), those that contribute the highest number of cases. The plan leaves behind regions considered at high risk, and that present higher incidence rates, like Ucayali and Madre de Dios, which have higher TB incidence than La Libertad and Ica which has been prioritized (144.5 and 128.8 vs. 65.1 and 106 respectively) [10]. Ucayali and Madre de Dios are also regions located in the Peruvian Amazon, with a high percentage of Amazonian indigenous population, but because of the focus on achieving the goals established as national indicators they were left out of the emergency plan. Indicators sensitive to intersectional categories that contribute to TB vulnerability, such as ethnicity, place of residence, economic status, must be created. This type of indicators would also allow moving from the current “risk approach”, where some individual characteristics (such ethnicity, place of residence) are presented as risk factors without an analysis of the structural factors contributing to the risk.

## Conclusions

The Peruvian health system needs to implement and adopt immediate measures to address the major weaknesses of the health system in rural areas if Peru is to fulfil the goals set by the End TB Strategy. This strategy must be accompanied by the development of a new set of indicators to assess the implementation of the TB programs. Our findings show that a fragmented health system is unprepared for delivering a health care that respects the various dimensions of the right to health, rendering marginalized populations, such as indigenous people vulnerable to preventable diseases and without access to treatment. Achieving control of the TB epidemic in our countries (and particularly in vulnerable populations such as indigenous communities) requires not only the implementation of new diagnostic tests and new TB drugs, but also a solid health system that internalizes the human rights approach in each of its activities and processes.

## Abbreviations

AAAAQ: AcceptabilityAvailability Affordability Adaptability and Quality
DDT: Detection of TB suspects, Diagnostics of those detected and Treatment those diagnosed
HIV: Human Immunodeficency Virus
MDR-TB: Multidrug-Resistant TB
MoH: Ministry of Health
PAT: People Affected by Tuberculosis
PNTBG: Peruvian National TB Guidelines
SIS: Seguro Integral de Salud
TB: Tuberculosis
WHO: World Health Organization
